# Structural insights of the p97/VCP AAA+ ATPase: How adapter interactions coordinate diverse cellular functionality

**DOI:** 10.1016/j.jbc.2023.105182

**Published:** 2023-08-22

**Authors:** Julian R. Braxton, Daniel R. Southworth

**Affiliations:** 1 Graduate Program in Chemistry and Chemical Biology, University of California, San Francisco, San Francisco, California, USA; 2 Department of Biochemistry and Biophysics and Institute for Neurodegenerative Diseases, University of California, San Francisco, San Francisco, California, USA

**Keywords:** p97, VCP, AAA+ ATPase, unfoldase, molecular chaperone, proteostasis, ERAD, autophagy, adapter, adaptor, cofactor, cryo-EM, protein structure prediction, AlphaFold

## Abstract

p97/valosin-containing protein is an essential eukaryotic AAA+ ATPase with diverse functions including protein homeostasis, membrane remodeling, and chromatin regulation. Dysregulation of p97 function causes severe neurodegenerative disease and is associated with cancer, making this protein a significant therapeutic target. p97 extracts polypeptide substrates from macromolecular assemblies by hydrolysis-driven translocation through its central pore. Growing evidence indicates that this activity is highly coordinated by “adapter” partner proteins, of which more than 30 have been identified and are commonly described to facilitate translocation through substrate recruitment or modification. In so doing, these adapters enable critical p97-dependent functions such as extraction of misfolded proteins from the endoplasmic reticulum or mitochondria, and are likely the reason for the extreme functional diversity of p97 relative to other AAA+ translocases. Here, we review the known functions of adapter proteins and highlight recent structural and biochemical advances that have begun to reveal the diverse molecular bases for adapter-mediated regulation of p97 function. These studies suggest that the range of mechanisms by which p97 activity is controlled is vastly underexplored with significant advances possible for understanding p97 regulation by the most known adapters.

p97 (also called valosin-containing protein, VCP, and Cdc48 in yeast) is an abundant and essential AAA+ (ATPases associated with diverse cellular activities) translocase present in all eukaryotic organisms. Since its discovery as a hexameric ATPase in 1990 (1), p97 has been associated with a wide and ever-expanding array of cellular functions, and has thus been dubbed a “cellular multitool” (2). These functions include many in protein and organellar homeostasis, membrane remodeling, protein trafficking, and chromatin-associated processes. p97 functions in these diverse processes by using the energy from ATP binding and hydrolysis to extract proteins from macromolecular complexes and membranes through unfolding, and is therefore considered a AAA+ “segregase”. In many pathways these substrates are unfolded by p97 to enable subsequent degradation by the 26S proteasome, which requires an unfolded or unstructured region to initiate processing (3, 4).

Central to p97 function are so-called “adapter” proteins that facilitate substrate unfolding and regulate p97 in various contexts. We define adapter proteins as those that enable a specific p97 function through direct interaction, facilitated by one or more p97-interacting domains. Notably, while p97 can hydrolyze ATP in the absence of adapters, these proteins appear required to bind substrate and initiate unfolding (5), and are therefore essential in all known p97 functions. There are over 30 known adapter proteins that have largely been identified by the presence of one or more conserved p97-interacting domains (6); in addition to adapters that recruit substrates, these include those that modify substrates, control p97 subcellular localization, alter ATPase activity, or regulate oligomeric state. Of note, in this review we do not differentiate between adapters that have been demonstrated to bind substrates and those that have not, given the possibility of latent substrate-binding activity (7). The term “adapter”, alternate spelling “adaptor”, and term “cofactor” are used interchangeably in the literature; for simplicity we exclusively use “adapter” here. Cellular studies have long established that specific adapters or sets thereof are important in certain processes, but the molecular interactions of most with p97 are uncharacterized, and thus little is known about what role(s) p97 plays in these processes. Recent research is shedding light on the structures of adapters and their mechanisms of action, though much remains to be discovered.

p97 is implicated in the etiology or progression of several diseases, underscoring its broad importance in cellular physiology. p97 has been investigated as a cancer target, as many cancers appear to particularly depend on p97 and other proteostasis factors in a phenomenon termed “non-oncogene addiction” (8, 9). To this end, many small molecule inhibitors of p97 have been developed, some of which have been evaluated in clinical trials (10). Regardless of clinical utility, some of these molecules are valuable p97 chemical probes, enabling acute and specific inhibition of p97 in cells and other experimental models (11). Furthermore, point mutations in p97 cause neurodegenerative diseases including inclusion body myopathy associated with Paget disease of bone and frontotemporal dementia (IBMPFD, also called multisystem proteinopathy), amyotrophic lateral sclerosis, and vacuolar tauopathy (12, 13, 14). Common to these disorders is the presence of abnormal protein deposits, implicating a defect in p97-dependent protein quality control as the cause of disease. Curiously, many disease-causing mutations appear to alter p97-adapter interactions, suggesting that perturbation of this network may contribute to disease (15, 16, 17, 18, 19) and underscoring the importance of adapters in p97 biology. Notably, due to the complexity of the p97 protein-protein interaction network, an emerging and attractive therapeutic modality for modulation of p97 function is the targeting of specific p97-adapter interactions with either small molecules or antibody fragments. Such tools could alter the interaction(s) perturbed in disease while sparing the broader p97 protein-protein interaction network, and are an active area of investigation (20, 21).

In this review, we discuss the current understanding of the structures and functions of p97 adapters. We highlight recent technological advances, including high-resolution cryo-EM structure determination and accurate artificial intelligence-based structure prediction that are enabling advances in understanding the molecular basis for adapter regulation of p97 function. These studies are beginning to reveal how diverse ensembles of adapters with numerous structural elements interact with p97 to direct function and cellular pathway. Structures of full-length adapters bound to p97 reveal that adapters frequently possess both conserved and novel p97-interacting domains which together coordinate p97 activity (22, 23, 24, 25). We suggest that future investigations will reveal additional motifs that bind and regulate p97, likely with distinct effects on p97 structure and function.

## p97-dependent cellular functions

**Figure 1:**
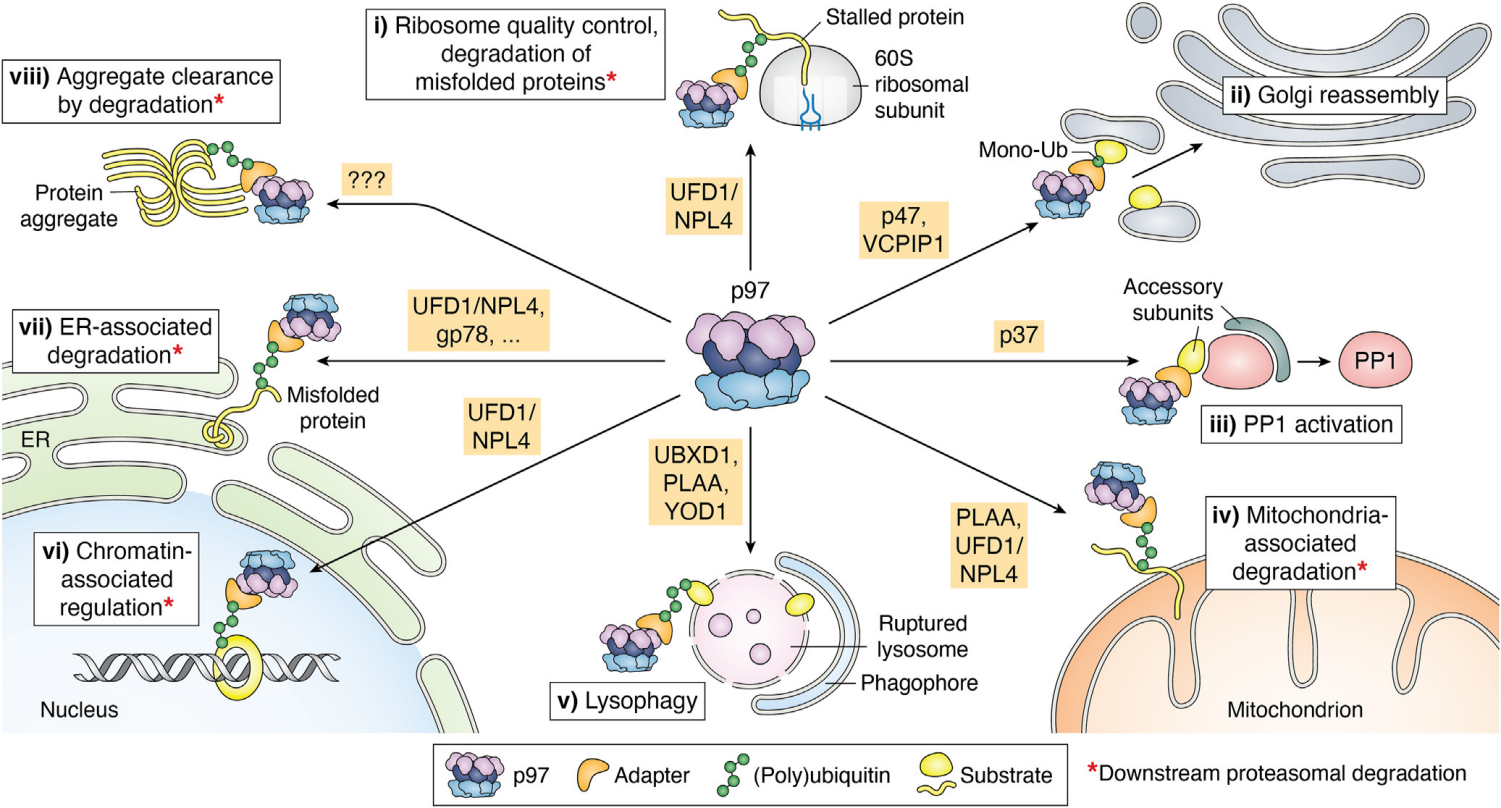
**Cellular functions of p97.** Overview of p97-dependent pathways, with associated adapters listed. p97 is colored by domain (NTD: *purple*, D1: *dark blue*, D2: *light blue*), and adapters are in *orange*. Substrates (*yellow*) are shown as *spherical* shapes to indicate folded, native states or as strands to indicate misfolded or unfolded states. Pathways where p97 activity promotes downstream proteasomal degradation are indicated (∗). p97-dependent processes are as follows (going clockwise from ribosome quality control): (i) as a component of ribosome quality control p97 with the adapter UFD1/NPL4 (UN) dislodges stalled substrates from 60S ribosomal subunits (28); (ii) during Golgi reassembly p97 with adapters p47 and VCPIP1 promotes fusion of postmitotic Golgi fragments through targeting of monoubiquitylated syntaxin 5 on the Golgi membrane (38, 39); (iii) in PP1 activation p97 with the p37 adapter extracts an inhibitory subunit (inhibitor 3) from an inactive PP1 precursor complex enabling formation of active holoenzymes (41, 42, 43); (iv) in mitochondria-associated degradation p97 with the adapters Doa1 (PLAA in humans) and UN extracts misfolded proteins from the outer mitochondrial membrane (101); (v) in lysophagy, extraction of (currently unknown) surface proteins on ruptured lysosomes by p97 with adapters UBXD1, PLAA, and YOD1 is required for phagophore formation (*blue*) and subsequent autophagic clearance (34); (vi) as an example of a chromatin-related function, after DNA double-strand break repair p97 with UN removes trapped Ku70/80 rings from intact DNA (33); (vii) in ER-associated degradation, p97 with UN, gp78, and many other adapters extracts misfolded proteins from the ER lumen (26, 102); (viii) in the clearance of tau fibrillar aggregates p97 may target ubiquitylated tau, though the relevant adapter(s) are unknown (45). ER, endoplasmic reticulum; NTD, N-terminal domain; PP1, protein phosphatase 1.

p97-facilitated segregation of substrates from macromolecular complexes is fundamental to many cellular processes (Fig. 1). p97 substrates are typically, though not exclusively, targeted for unfolding by the attachment of ubiquitin chains. p97 activity can be classified into two groups: facilitating proteasomal degradation of substrates to maintain protein homeostasis, and regulating cellular processes. Regarding degradation, many proteins destined for the 26S proteasome need to be extracted from large assemblies or cellular compartments in order to gain access to proteolytic machinery; ubiquitylation targets these substrates to both p97 and the proteasome. Well-known examples of degradative processes are endoplasmic reticulum-associated degradation (ERAD) (26, 27), in which misfolded proteins need to be dislodged from the endoplasmic reticulum (ER) membrane, and ribosome-associated quality control (28, 29), in which nascent polypeptides need to be extracted from stalled ribosomes. Because it enables degradation of many aberrant proteins in the cell, p97 is central to protein quality control and cellular stress response. p97 inhibition, through chemical or genetic means, therefore causes severe proteostasis defects (30).

The regulatory processes facilitated by p97 substrate unfolding are less understood, but are nonetheless critical elements of p97 function. For example, p97 facilitates disassembly of many multiprotein complexes from chromatin, including the replicative helicase (31, 32) and the double-strand break sensor Ku70/80 (33), enabling completion of DNA replication and double-strand break repair, respectively. Remodeling of surface proteomes by p97 also appears to be important for organellar homeostasis: p97 activity enables clearance of damaged mitochondria and lysosomes through macroautophagy, termed mitophagy and lysophagy, respectively (34, 35, 36, 37). Additionally, p97 enables endolysosomal trafficking of some proteins (17), and facilitates Golgi and ER reassembly by promoting membrane fusion (38, 39), potentially through a similar regulatory mechanism. Similar to substrates destined for proteasomal degradation, substrates involved in regulatory processes are typically targeted to p97 by the presence of ubiquitin. However, this is not exclusively the case, as it has recently been demonstrated that p97 facilitates protein phosphatase 1 holoenzyme formation by extracting an inhibitory subunit from a precursor complex without ubiquitin conjugation (40, 41, 42, 43). Importantly, some substrates in p97-dependent regulatory pathways are also degraded by the proteasome, as in the extraction of Ku80 from DNA (44), indicating a functional overlap with proteostatic pathways. Finally, the mechanisms of some p97-dependent processes, such as clearance of tau fibrils (13, 45), are largely unknown, and there are likely more p97-dependent processes yet to be discovered.

## Mechanism of p97 substrate unfolding

**Figure 2:**
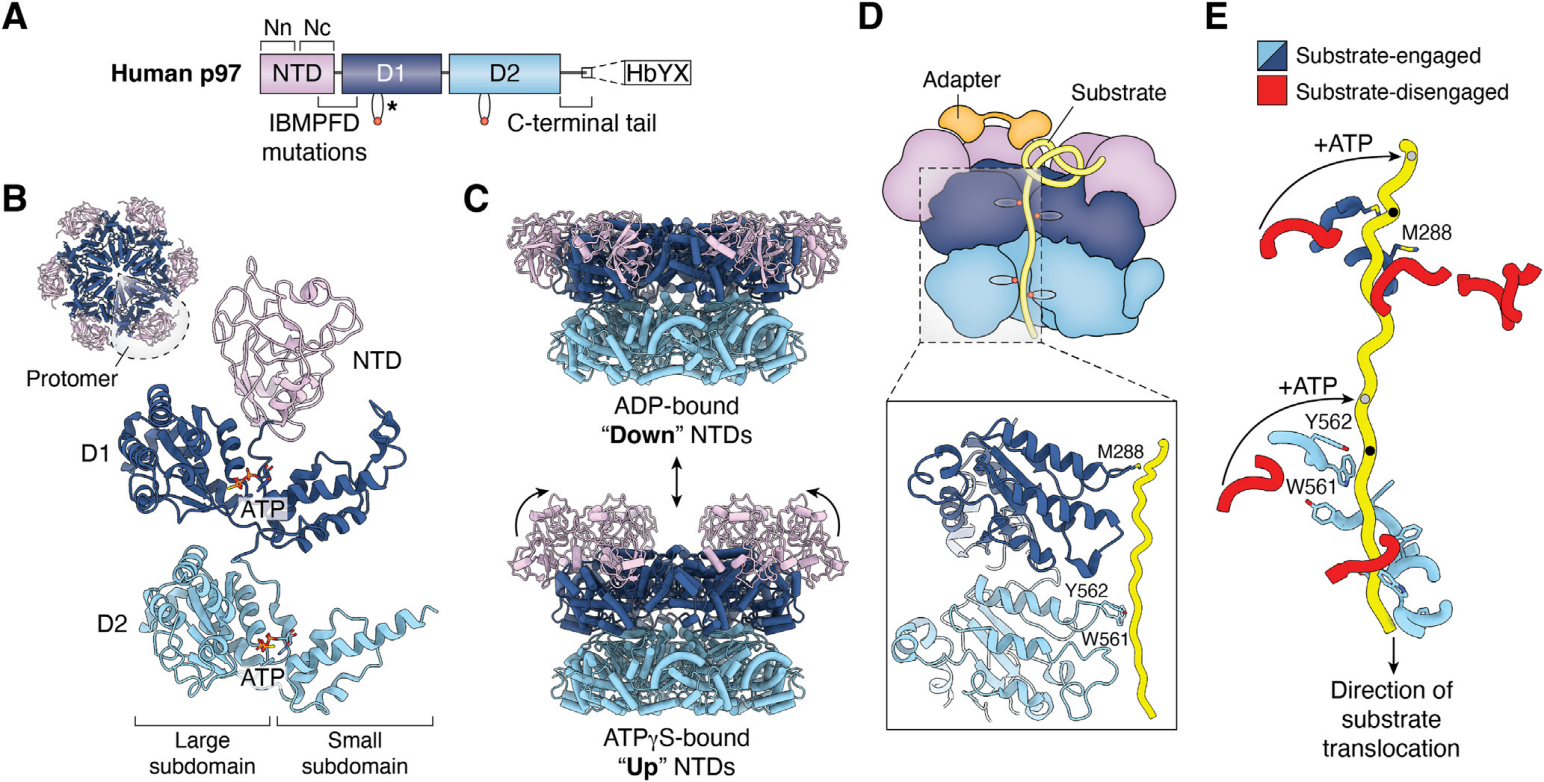
**Structure and substrate threading mechanism of p97.***A*, domain schematic of human p97 colored by domain (NTD: *purple*, D1: *dark blue*, and D2: *light blue*). Location of pore loops in D1 and D2 are shown; degenerate residues in D1 are indicated (∗). The two lobes of the NTD (N- and C-terminal lobes, Nn and Nc) are indicated, as is the location of most IBMPFD mutations, namely the NTD-D1 interface. The HbYX motif at the end of the C-terminal tail is also shown. *B*, protomer of ATPγS-bound p97 from an intact hexamer (PDB 5FTN) (52), colored as in (*A*). A downscaled top view of the full hexamer is also shown, with one protomer circled to delineate protomer boundaries. *C*, side views of ADP-bound (*top*, PDB 5FTK) and ATPγS-bound (*bottom*, PDB 5FTN) p97 hexamers (52), showing rotation and elevation of the NTDs above the D1 ring in the ATPγS-bound state. *D*, illustration of adapter-mediated substrate threading through the p97 central pore, with D1 and D2 pore loops shown. The adapter is colored in *orange*, and the substrate in *yellow*. Below, an enlarged view of pore loop contacts is shown (PDB 6OA9) (23). *E*, view of substrate in the Cdc48 channel (PDB 6OA9) (23), showing a spiral arrangement of pore loops in D1 and D2. Pore loops engaged with substrate are shown in *blue*; those not engaged are in *red*. The highest substrate contacts in D1 and D2 are marked by *black dots*; hydrolysis and subsequent ATP binding by the disengaged protomers is proposed to drive translocation by two amino acid steps whereby conformational changes enable pore loop engagement with the next site along the substrate (*gray dots*). IBMPFD, inclusion body myopathy associated with Paget disease of bone and frontotemporal dementia; NTD, N-terminal domain; PDB, Protein Data Bank.

p97 contains two AAA+ domains (D1 and D2) that bind and hydrolyze ATP (Fig. 2, *A* and *B*). As in all AAA+ proteins, nucleotide binding in both AAA+ domains occurs in a cleft at the interface of constituent large and small subdomains, and hydrolysis is facilitated by Walker A and B and Sensor-I and -II motifs, as well as *trans*-acting arginine fingers from an adjacent protomer (46). p97 additionally features an N-terminal domain (NTD) and a flexible C-terminal (CT) extension, which are the primary sites of adapter interaction. At the end of the CT tail is a HbYX (hydrophobic, Tyr, any amino acid) motif that in other proteins enables interaction with the 20S proteasome, though no such interaction has been definitively identified in eukaryotic p97 homologs (47). Initial structures revealed that p97 protomers assemble to form homohexamers, which enclose a central channel (48, 49). As in other AAA+ proteins, this channel features pore loops in D1 and D2 that were hypothesized to interact with substrate proteins (50). Curiously, while D2 pore loop residues are aromatic, as is typical, the D1 residues are degenerate, perhaps indicating a distinct function of the D1 domain. Supporting this hypothesis, the position of the NTDs with respect to the AAA+ core was observed to be dependent on D1 nucleotide occupancy; in this mechanism, ADP binding induces the formation of a “down” conformation of the NTD, coplanar with the D1 ring, while ATP binding causes the NTDs to rotate above the D1 ring in a more flexible “up” state (Fig. 2*C*) (51, 52). NTD conformation strongly influences binding affinity of some adapters (15, 17, 18, 19, 53, 54); thus, a major function of D1 appears to be the control of adapter interactions through nucleotide state.

A significant advance in understanding p97 function came from *in vitro* experiments done by Rapoport *et al.*, focusing on the yeast p97 homolog, Cdc48, in the ERAD pathway (26). This study demonstrated that luminal ERAD substrates are ubiquitylated by a membrane-bound E3 ligase, which results in the recruitment of Cdc48. Cdc48 ATPase activity dislodges substrate from the E3, allowing for proteasomal degradation. This molecular mechanism was further elucidated in an additional landmark study by the Rapoport group, showing that Cdc48 unfolds ubiquitylated substrates and fully translocates them through its central channel in an ATPase-dependent manner (55). This work established that Cdc48 and its close homologs in higher eukaryotes indeed unfold substrates as do other AAA+ translocases. Breakthrough structures of substrate- and adapter-bound complexes achieved by the Rapoport and Shen groups then provided direct observation of Cdc48-mediated substrate unfolding (23, 56). These structures revealed that p97 forms right-handed spirals that enable the interaction of pore loops residues along an unfolded substrate (Fig. 2, *D* and *E*). Surprisingly, the degenerate pore loop residues in D1 (including Met288) were also identified to contact substrate in both structures, expanding the known modes of pore loop-substrate interactions in AAA+ proteins. The NTDs in these structures are in the “up” conformation, indicating that this is the conformation active for substrate unfolding. Indeed, IBMPFD mutations that increase the frequency of the “up” conformation enhance this activity (Fig. 2*A*) (18). Substrate translocation is proposed to occur in a processive, hand-over-hand mechanism in which ATP hydrolysis and subsequent binding of ATP to the lowest protomer causes its movement to the top of the hexamer, advancing substrate contact by ∼two amino acids. This mechanism, in part, arose from previous substrate-bound structures of related AAA+ complexes including Hsp104, ClpB, and YME1, and is thought to be conserved among all AAA+ translocases (57, 58, 59).

## Adapters are defined by conserved p97-interacting domains

**Figure 3:**
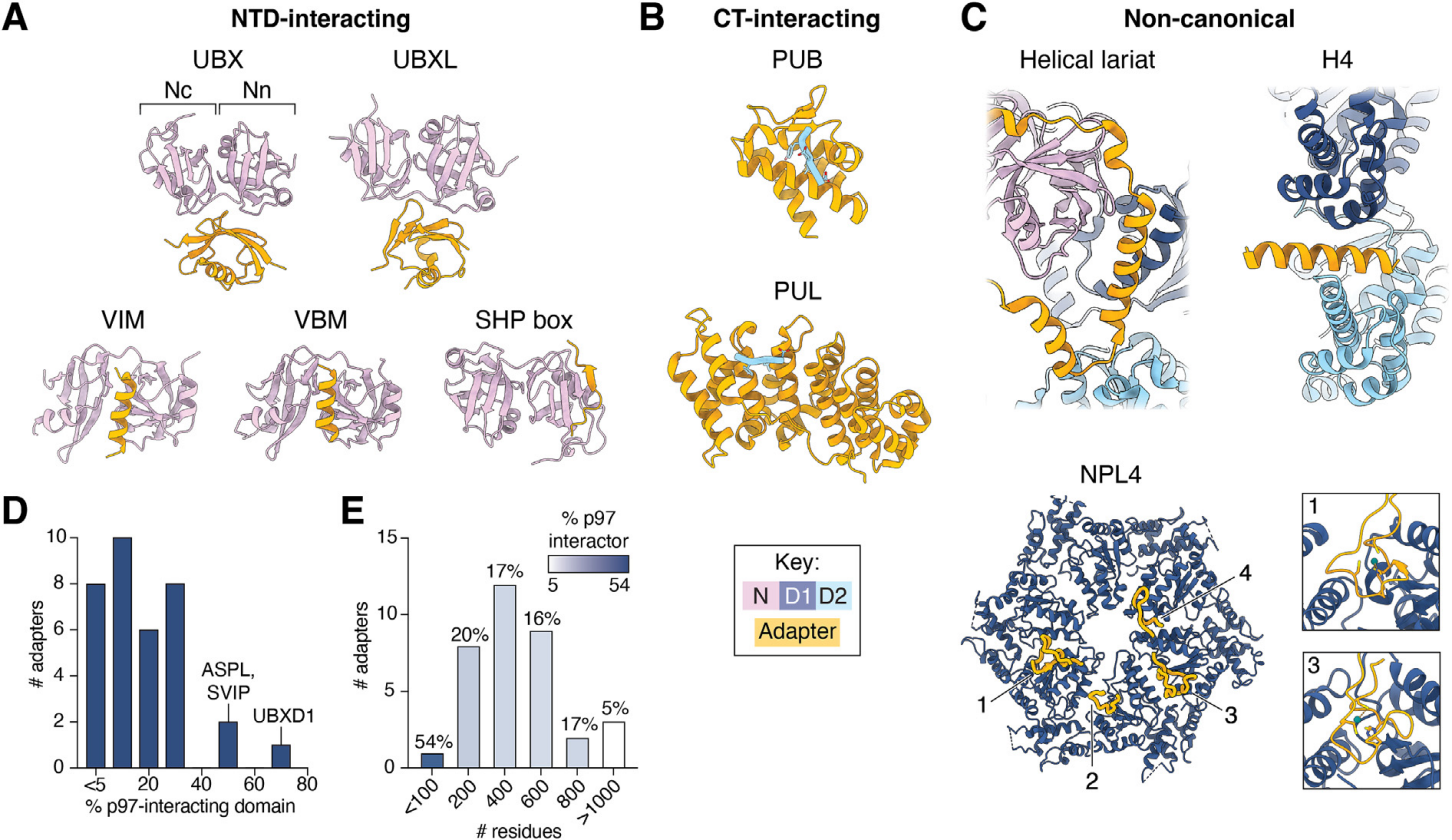
**Architecture and interaction modes of p97 adapters.** Structures of individual p97-interacting domains bound to the p97 NTD, CT tail, or other sites are shown, highlighting the diversity of adapter interaction modes. *A*, structures of the p97 NTD bound to NTD-interacting domains. The p97 NTD lobes are indicated in the UBX-bound structure. PDB IDs: UBX: 5X4L (63), UBXL: 4KDI (66), VIM: 3TIW (67), VBM: 5EPP (68), and SHP box: 5GLF (69). *B*, structures of the HbYX motif at the end of the p97 CT tail bound to CT-interacting domains. PDB IDs: PUB: 2HPL (73) and PUL: 3EBB (71). *C*, structures of noncanonical p97-interacting domains bound to p97 or a homolog thereof. PDB IDs: helical lariat: 8FCL (24), H4: 8FCR (24), and NPL4: 6OA9 (23). The four interacting motifs in NPL4 are numbered counterclockwise: zinc finger 1 (ZF1, 1), N-terminal bundle (NTB, 2), zinc finger 2 (ZF2, 3), and β-strand finger (4). Enlarged views of ZF1 and ZF2 are shown at right; Zn^2+^ is colored in *teal*. *D*, histogram of the proportion of adapters corresponding to p97-interacting domains (determined by % of total sequence length). *E*, histogram of adapter length. Bars are colored by average % p97 interactor of adapters in that bin (determined by % of total sequence length). The color key indicates p97 domains, as in Figure 2, and adapters. CT, C-terminal; NTD, N-terminal domain; PDB, Protein Data Bank; PUB, peptide:*N*-glycanase and UBA or UBX-containing proteins; PUL, PLAP, Ufd3p, and Lub1p; UBX, ubiquitin regulatory X; UBXL, UBX-like; VBM, VCP-binding motif; VIM, VCP-interacting motif.

Adapters of p97 exhibit striking structural and functional diversity (Fig. 3 and Table 1). These proteins are typically identified by the presence of one or more domains that interact at specific NTD and CT sites on p97. Given the conservation of many of these domains, the number of known or predicted adapters has been expanded through bioinformatic analysis of eukaryotic genomes (60, 61). Many adapters are modular assemblies of domains connected by unstructured linkers of unknown function, suggesting that conformational flexibility is an important aspect of p97-adapter interactions. While NTD and CT tail interactions are well-described, other interaction modes are now being reported, highlighting the many ways in which adapters bind p97.

**Table 1:** Human p97 adapters

Common protein name	Protein	Gene	UniProt ID	p97-interacting domain											Enzymatic activity	Substrate binding?	Total length (aa)	Residues in p97-interacting domain(s)	% p97 interacting
				UBX	UBXL	VIM	VBM	SHP	PUB	PUL	H1/H2	H4	Lariat	NPL4					
AIRAPL	AN1-type zinc finger protein 2B	*ZFAND2B*	Q8WV99			•									N/A	Yes	257	14	5.4
ANKZF1	Ankyrin repeat and zinc finger domain-containing protein 1	*ANKZF1*	Q9H8Y5			•									Peptidyl-tRNA hydrolase	Yes	726	16	2.2
ASPL (UBXD9)	Tether containing UBX domain for GLUT4	*ASPSCR1* (*UBXN9*)	Q9BZE9	•	•			•					•		N/A	No	553	260	47.0
Ataxin-3	Ataxin-3	*ATXN3*	P54252				•								Deubiquitinase	Yes	361	27	7.5
CHIP	E3 ubiquitin-protein ligase CHIP	*STUB1*	Q9UNE7				•								E3 ubiquitin ligase	Yes	303	6	2.0
Derlin-1	Derlin-1	*DERL1* (*DER1*)	Q9BUN8					•							N/A	Yes	251	13	5.2
Derlin-2	Derlin-2	*DERL2* (*DER2*)	Q9GZP9					•							N/A	Yes	239	13	5.4
FAF1 (UBXD12)	FAS-associated factor 1	*FAF1* (*UBXN3A*)	Q9UNN5	•											N/A	Yes	650	78	12.0
FAF2 (UBXD8)	FAS-associated factor 2	*FAF2* (*UBXN3B*)	Q96CS3	•											N/A	Yes	445	81	18.2
gp78	E3 ubiquitin-protein ligase AMFR	*AMFR*	Q9UKV5			•									E3 ubiquitin ligase	Yes	643	19	3.0
HOIP	E3 ubiquitin-protein ligase RNF31	*RNF31*	Q96EP0						•						E3 ubiquitin ligase	Yes	1072	78	7.3
HRD1	E3 ubiquitin-protein ligase synoviolin	*SYVN1*	Q86TM6				•								E3 ubiquitin ligase	Yes	617	13	2.1
NPL4	Nuclear protein localization protein 4 homolog	*NPLOC4*	Q8TAT6		•									•	N/A	Yes	608	167	27.5
NUB1	NEDD8 ultimate buster 1	*NUB1*	Q9Y5A7				•								N/A	Yes	615	37	6.0
OTU1 (YOD1)	Ubiquitin thioesterase OTU1	*YOD1*	Q5VVQ6		•										Deubiquitinase	Yes	348	77	22.1
p37	UBX domain-containing protein 2B	*UBXN2B*	Q14CS0	•				••							N/A	Yes	331	88	26.6
p47	NSFL1 cofactor p47	*NSFL1C* (*UBXN2C*)	Q9UNZ2	•				••							N/A	Yes	370	89	24.1
PLAA (PLA2P, PLAP, UFD3)	Phospholipase A-2-activating protein	*PLAA*	Q9Y263							•					N/A	Yes	795	250	31.4
PNGase	Peptide-N(4)-(N-acetyl-beta-glucosaminyl)asparagine amidase	*NGLY1*	Q96IV0						•						Deglycosylase	Yes	654	67	10.2
RHBDL4	Rhomboid-related protein 4	*RHBDD1*	Q8TEB9				•								Protease	Yes	315	15	4.8
SAKS1	UBX domain-containing protein 1	*UBXN1* (*SAKS1*)	Q04323	•											N/A	Yes	297	85	28.6
SPRTN (DVC1)	DNA-dependent metalloprotease SPRTN	*SPRTN*	Q9H040					•							Protease	Yes	489	12	2.5
SVIP	Small VCP/p97-interacting protein	*SVIP*	Q8NHG7			•						•			N/A	No	77	42	54.5
TEX264	Testis-expressed protein 264	*TEX264*	Q9Y6I9					•							N/A	Yes	313	13	4.2
UBE4B (UFD2)	Ubiquitin conjugation factor E4 B	*UBE4B*	O95155				•								E3 ubiquitin ligase	Yes	1302	13	1.0
UBXD1	UBX domain-containing protein 6	*UBXN6*	Q9BZV1	•		•			•		•	•	•		N/A	Unconfirmed	441	303	68.7
UBXD2 (Erasin)	UBX domain-containing protein 4	*UBXN4*	Q92575	•											N/A	No	508	77	15.2
UBXD3	UBX domain-containing protein 10	*UBXN10*	Q96LJ8	•											N/A	No	280	75	26.8
UBXD4	UBX domain-containing protein 2A	*UBXN2A*	P68543	•				•							N/A	Yes	259	86	33.2
UBXD5 (Socius)	UBX domain-containing protein 11	*UBXN11* (*SOC*)	Q5T124	•				•							N/A	Yes	520	87	16.7
UBXD6	UBX domain-containing protein 8	*UBXN8*	O00124	•											N/A	No	270	75	27.8
UBXD7	UBX domain-containing protein 7	*UBXN7*	O94888	•											N/A	Yes	489	78	16.0
UFD1	Ubiquitin recognition factor in ER-associated degradation protein 1	*UFD1* (*UFD1L*)	Q92890					••							N/A	Yes	307	24	7.8
VCPIP1 (VCIP135)	Deubiquitinating protein VCPIP1	*VCPIP1*	Q96JH7		•										Deubiquitinase	Yes	1222	77	6.3
VIMP	Selenoprotein S	*SELENOS*	Q9BQE4			•									N/A	No	189	51	27.0

Dots indicate the presence of the above p97-interacting domain in each adapter, with the number of dots representing the number of elements in each sequence.

The NTD is the most common site of adapter interaction, and binds five conserved classes of p97-interacting domains (Fig. 3*A*). The most prevalent of these are the ubiquitin regulatory X (UBX) and UBX-like (UBXL) domains, which are ∼80 residue globular domains structurally similar to ubiquitin that bind the NTD in the cleft formed between its two constituent lobes (N-terminal, Nn, and C-terminal, Nc) (62). UBX domains interact with the NTD using an arginine in the β1 strand and a tripeptide motif located in a loop (S3/S4) between β3 and β4 with consensus sequence Phe-Pro-Arg (62, 63, 64, 65). UBXL domains bind in a similar orientation, though the residues mediating the interactions are distinct (66). The next class of NTD-interacting domains are the linear VCP-interacting (VIM) and VCP-binding motifs, which bind by projecting charged residues into the same Nn-Nc cleft as do UBX(L) domains (60, 67, 68). Finally, the SHP box, also called binding site 1, is a short peptide of ∼8 residues that adopts a β-strand conformation with the β-sheet of the Nn lobe (69). In contrast to the large number of NTD-interacting domains, only two domains have been identified to bind the p97 CT tail, the peptide:*N*-glycanase and UBA or UBX-containing proteins (PUB) and PLAP, Ufd3p, and Lub1p (PUL) domains (Fig. 3*B*) (70, 71, 72). Both interact with the last residues of this tail (804-Leu-Tyr-Gly-806, the HbYX motif), and thus localize adapters containing these domains to the D2 face of p97. Notably, phosphorylation of Tyr805 is a major mechanism by which PUB/PUL interactions are regulated, as this posttranslational modification abolishes interaction with the CT tail (70, 73).

In addition to highly conserved p97-interacting domains, some adapters harbor noncanonical p97-interacting elements that enable distinct mechanisms of modulating p97 structure and function (Fig. 3*C*). These include the helical lariat of ASPL and UBXD1, which is a four-helix insertion in their UBX domains that encircles the NTD and causes the disruption of p97 interprotomer D1-D1 contacts (24, 74). Similarly, the H4 motif found in UBXD1 (and predicted in SVIP) appears to disrupt D2-D2 contacts and inhibit p97 ATPase activity (24). Finally, NPL4 features four extensions that anchor this adapter directly above the p97 central channel using D1 interactions, facilitating substrate threading through the central channel (22). Additional p97-interacting domains have yet to be structurally characterized, including the H1/H2 region of UBXD1, which interacts with the NTD-D1 interface (19, 75).

p97-interacting domains frequently account for only a small percentage of the overall length of adapters (Fig. 3*D*). Rather, most known p97 adapters contain additional domains that enable direct interaction with substrates to facilitate their unfolding or posttranslational modification, or contain unannotated regions. In addition to those that simply bind substrates (as with ubiquitin-associated, UBA, domains that bind ubiquitin and are found in many adapters), many of these domains modify ubiquitin chains on substrate, and include deubiquitinases (as in YOD1, Ataxin-3, and VCPIP1) and ubiquitin ligases (as in HRD1, HOIP, and UBE4B); other posttranslational modifications are enabled by deglycosylases (PNGase) and proteases (SPRTN) (reviewed in (2)). However, some adapters (20%, 7/35) lack these additional domains, and their role in modulating p97 activity is therefore less understood. Moreover, 26% of known adapters (9/35) have multiple domains that interact with p97, though why more than one such domain is required in these adapters is not known in many cases. Indeed, the (predicted) ordered regions of some adapters, namely UBXD1, ASPL, and SVIP, appear to be composed exclusively of these domains (Fig. 3*D*). In stark contrast to others, ∼50% or greater of the sequence of these three adapters are accounted for by p97-interacting domains, with UBXD1 having the highest percentage (∼70%) due to its six p97-interacting domains. Furthermore, adapter length is loosely anticorrelated with % p97-interactor content: the minimal adapter SVIP (77 residues) is composed of ∼50% p97-interacting regions, the middle ∼90% of adapters are composed of ∼20% of such regions, and the three longest adapters are composed of ∼5% of such regions (Fig. 3*E*). Together, these observations underscore the complexity of adapter domain organization.

Specific adapters or combinations thereof are linked to specific p97-dependent functions. For example, many p97 substrates are recognized through attachment of polyubiquitin chains, and the ubiquitin-binding adapter UFD1/NPL4 is therefore linked to numerous p97 processes including ERAD, ribosome-associated quality control, chromatin-associated degradation, and others (26, 28, 76, 77). In contrast, some adapters appear to facilitate more specialized processes. For example, the SPRTN metalloprotease mediates recruitment of p97 to sites of DNA damage, which facilitates the extraction of specific DNA damage response proteins and may limit the number of mutations introduced (78, 79, 80). Of note, combinations of substrate-binding and nonbinding adapters appear to cooperate in certain p97 processes, including YOD1 and PLAA (binding) and UBXD1 (nonbinding/not definitively demonstrated) in lysophagy (34), suggesting the functions of these two classes are not mutually exclusive.

The complexity of the p97 adapter network, already significant in lower eukaryotes, is even more apparent in humans. For example, SEP (Shp1, eyes closed, and p47)-domain proteins are among the most studied p97 adapters due to their abundance and affinity for p97 (39). Only one SEP-domain adapter, Shp1, exists in yeast, whereas humans have four, all of which appear to have distinct functions. Moreover, some adapters such as UBXD1 are only present in higher eukaryotes, implying a more sophisticated range of p97 regulation and function. Of note, it is possible that there are p97 adapters yet to be discovered due to the presence of currently uncharacterized p97-interacting domains. Finally, the logic of adapter interactions in certain p97 processes is intriguing. For example, many effectors of ERAD directly interact with p97, including channel-forming Derlins (81), the ER membrane-resident E3 ubiquitin ligase gp78 (82), and the ER membrane-resident adapter VIMP (83, 84). Clearly, localization of p97 to the ER membrane is critical, but why all of these adapters need p97-interacting domains is unclear.

## High-resolution structures of intact p97-adapter complexes reveal novel regulatory mechanisms and interaction modes

Structural interrogation of adapter proteins has until recently been limited to structures of isolated p97-interacting domains (with or without p97 or truncations thereof) and low-resolution cryo-EM reconstructions. While these studies have provided foundational insight into p97-adapter interactions and generated preliminary models of adapter-mediated regulation of p97, a major limitation has been the lack of full-length p97-adapter structures. Excitingly, in the past few years, much progress has been made toward the goal of structural characterization of intact p97 complexes. Many of these discoveries have relied on single-particle cryo-EM, which is well-suited for these efforts given the significant conformational and compositional heterogeneity inherent in p97 complexes. While some advances have been made toward a complete structural understanding of p97-adapter interactions, it is likely that this approach will continue to enable similar breakthroughs. Notable developments are discussed below.

### Substrate unfolding by UFD1/NPL4 and SEP-domain adapters

**Figure 4:**
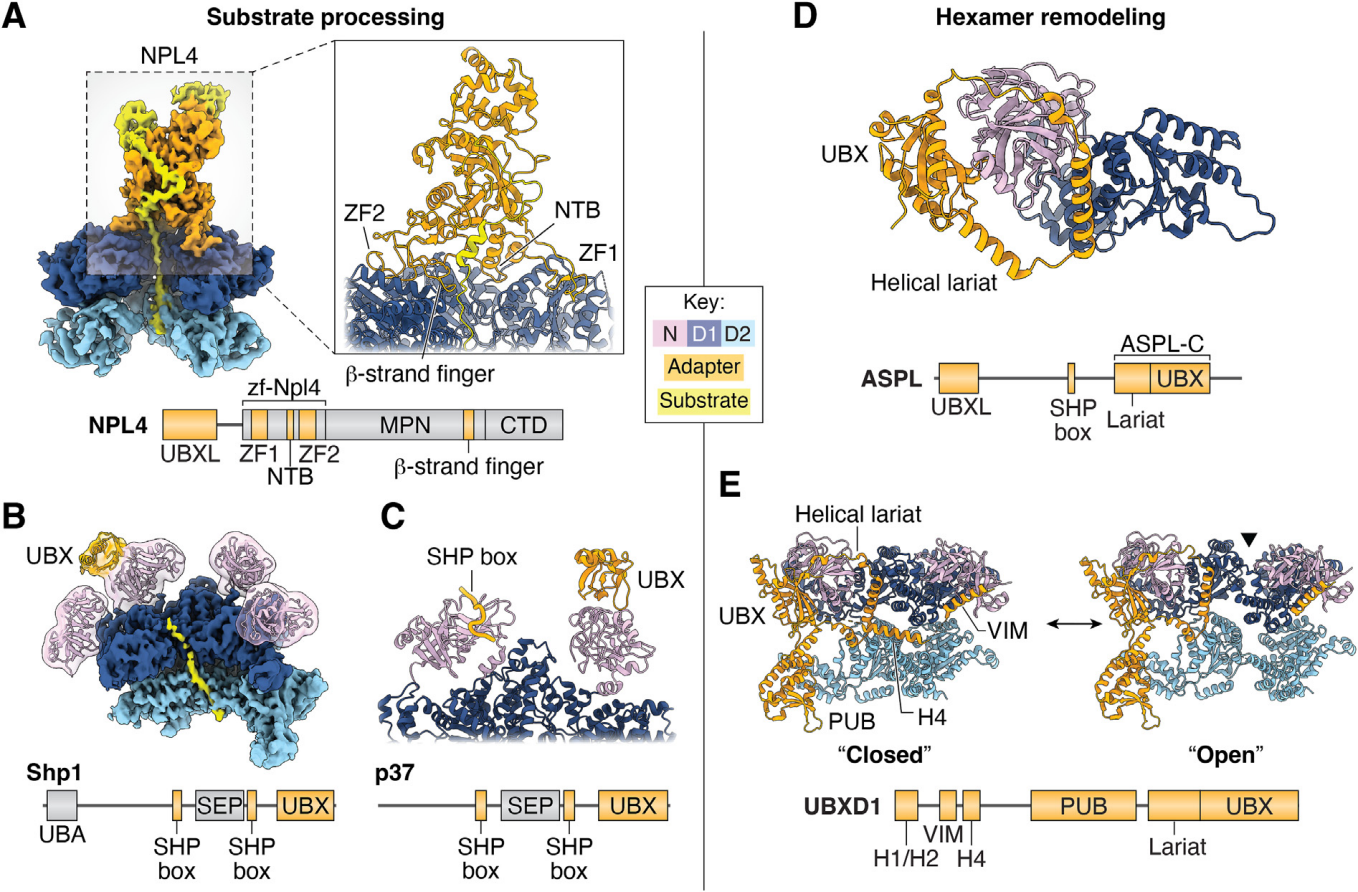
**Structures of intact p97-adapter complexes.** Structures of p97-adapter complexes involved in substrate processing (*left*) or hexamer remodeling (*right*). Domain colors are indicated by the key. Domain schematics (not to scale) of each adapter are shown below the corresponding structure, with p97-interacting domains colored in *orange* and other domains in *gray*. *A*, (*left*) cryo-EM structure of yeast Cdc48 bound to the UFD1/NPL4 adapter (PDB 6OA9) (23), unfolding an ubiquitylated substrate and threading it through the Cdc48 central channel. (*Right*) Enlarged view of contacts made between NPL4 and Cdc48. *B*, cryo-EM structure of Cdc48 bound to substrate and the Shp1 adapter (PDB 6OPC) (56). A filtered transparent map and model of the NTDs and UBX is shown over the sharpened map of the D1, D2, and substrate. *C*, cryo-EM structure of a SHP box and UBX domain of p37 bound to adjacent NTDs of an actively processing p97 complex (PDB 8B5R) (43). *D*, crystal structure of an ASPL truncation construct containing the UBX and helical lariat domains (ASPL-C) bound to a human p97 construct (PDB 5IFS) (74). *E*, cryo-EM structures of human p97 bound to UBXD1 (closed: PDB 8FCR, open: PDB 8FCM) (24), showing separation of adjacent p97 protomers coordinated by multiple UBXD1 contacts. The hexamer seam is indicated in the open state by a *black triangle*. NTB, N-terminal bundle; NTD, N-terminal domain; PDB, Protein Data Bank; UBX, ubiquitin regulatory X; ZF, zinc finger.

How adapters enable unfolding of p97 substrates has been an area of active study. Direct observation of this activity has only recently been achieved, in studies using Cdc48 and either the UFD1/NPL4 or Shp1 adapters (23, 56). These studies, both published in 2019, reveal many insights about the mechanism of p97 substrate unfolding and the role of adapters therein (Fig. 4, *A* and *B*).

While the mechanism of substrate translocation appears conserved among all AAA+ proteins (Fig. 2*E*), the roles of adapter proteins in enabling this activity are distinct. The heterodimeric UFD1/NPL4 adapter is among the best structurally characterized due to its stable tower-like structure on the D1 face of p97. NPL4 binds K48-linked ubiquitin molecules in folded and unfolded states using its MPN and CT domains; unfolded ubiquitin is bound in a conserved groove positioned directly above the entrance to the p97 central channel. Interactions by the UT3 domain of UFD1 also contribute to ubiquitin binding. In agreement with a previous substrate-free Cdc48/UFD1/NPL4 structure (22), interactions with the p97 hexamer are extensive, and involve conserved and unique interactions that could not be completely anticipated based on homology to other proteins or domains thereof (Fig. 4*A*). NPL4 uses two zinc fingers, an N-terminal bundle, and a β-strand finger to anchor onto the D1 ring, as well as a UBXL domain that binds the p97 NTD. The UT6 domain of UFD1 harbors two SHP boxes that also bind NTDs, though they, and the UBXL, are not well-resolved due to the flexibility of the NTDs in the up state. Together these structures reveal an intimate and highly coordinated arrangement of p97- and substrate-interacting domains that enable unfolding of ubiquitylated substrates.

In contrast to the multifaceted interactions of UFD1/NPL4 with p97 and substrate, SEP-domain adapters (including yeast Shp1 and human homologs p37 and p47) are considerably simplified. Shp1 has a UBA domain that interacts with ubiquitin, as well as a UBX and two SHP boxes that bind p97 NTDs. Immunoprecipitation and cryo-EM imaging of Shp1 from yeast in the presence of ADP-BeFx yielded substrate-bound Cdc48 complexes, in which the only non-Cdc48 density was the unfolded substrate in the central channel and the UBX of Shp1 (Fig. 4*B*) (56). This suggests that Shp1 flexibly associates with Cdc48 and substrate, rather than adopting a stable structure as with UFD1/NPL4. Excitingly, a recent publication reporting additional Cdc48-Shp1 structures identified a new density proximal to the D1 ring; using machine learning-based structure prediction, this density was assigned as a new p97-interacting motif in Shp1 (the D1-binding motif, D1B) (25). Substrate-bound (43, 85) and -unbound (86) structures of p97 complexes with human SEP-domain adapters further confirm the flexible association of these adapters with p97. Notably, one of these structures, that of p97-p37 extracting an inhibitory subunit from a protein phosphatase 1 complex, features the first observation of a SHP box from a full-length adapter bound to any p97 homolog (43). In this structure, the SHP box and UBX are bound to adjacent NTDs (Fig. 4*C*), though whether this arrangement is conserved in other SEP-domain adapters is unknown.

### Hexamer remodeling by ASPL and UBXD1

In addition to directly promoting substrate unfolding or modification, other adapters appear to have more regulatory roles in p97 biology, including ASPL and UBXD1 (Fig. 4, *D* and *E*). ASPL has a UBX, UBXL, and SHP box, and was identified to dissociate p97 hexamers into smaller oligomers and monomers (74). Truncation analysis revealed that the region responsible for this activity was the UBX domain and an uncharacterized region inserted therein termed the helical lariat. X-ray crystallography of p97 and an ASPL construct containing these elements revealed that the UBX domain interacts with the p97 NTD in a manner conserved among all known UBX proteins, but the helical lariat structure completely encircles this NTD and contacts the associated D1 through unprecedented interactions (Fig. 4*D*) (74). Though a definitive cellular role of ASPL-mediated p97 disassembly is lacking, a recent study reported that this disassembly enables methylation of an otherwise inaccessible lysine residue by METTL21D (also known as VCPKMT), which could potentially alter p97 activity (87). This work reveals that p97 structure is regulated through diverse and unanticipated mechanisms.

The complexity of adapters is also well-illustrated by our recent study of the UBXD1 adapter (24). UBXD1 is implicated in several p97-dependent processes including clearance of damaged mitochondria and lysosomes through macroautophagy, as well as endolysosomal sorting of ubiquitylated cargo proteins (17, 34, 35, 75, 88). As with ASPL, UBXD1 does not have any conserved domains that bind to or modify substrates, making its role in these processes unclear. However, coordination with substrate-binding adapters in lysophagy (including the deubiquitinase YOD1 and ubiquitin-binding adapter PLAA) is suggestive of a substrate-related function. UBXD1 possesses many p97-interacting regions including conserved VIM, UBX, and PUB domains. Cryo-EM structures of p97-UBXD1 complexes revealed that UBXD1 causes a striking separation of two adjacent p97 protomers, coordinated by its many p97-interacting domains (Fig. 4*E*). In addition to the VIM, UBX, and PUB domains, other regions including a previously unannotated helical lariat and helix (H4) adjacent to the VIM cause the disruption of inter-protomer D1 and D2 contacts, respectively. Of note, UBXD1 interactions with p97 are the most extensive of any characterized adapter, and likely of any adapter in general (Fig. 3*D*). Though the purpose of the structural remodeling caused by UBXD1 is unknown, possible explanations include facilitating the release of stalled substrates or enabling translocation of substrates too large for ordinary initiation. Surprisingly, a recent study reported a ubiquitin-binding activity of the UBXD1 UBX domain (7), further supporting a substrate-related role for this adapter. This observation underscores the enigmatic and novel functions characteristic of adapters as a whole.

## Technological advances enabling structural studies of adapters

Analysis of full-length p97-adapter complexes has been facilitated by many technologies, and will likely continue through the use of emerging ones. Perhaps most notably, single-particle cryo-EM has been pivotal in many of the studies discussed above, enabling structure determination of UFD1/NPL4-, Shp1-, p37, p47, and UBXD1-bound complexes. In addition to single-particle cryo-EM, other established technologies that have been successfully used in these and other studies include artificial intelligence-enabled protein structure prediction, rapid immunoprecipitation of p97 complexes, and mass spectrometry-based proteomics. The potential advantages of each technology for the study of p97-adapter interactions in general are discussed below.

It is widely appreciated that improved microscope design, direct electron detectors, and maximum-likelihood based classification algorithms have enabled the so-called “resolution revolution” in single-particle cryo-EM, making it the current method of choice for structural investigation of multiprotein complexes (89). Beyond these generally applicable developments, there are challenges specific to p97-adapter structure determination that make advances in cryo-EM software particularly enabling. First, p97 complexes are often characterized by a relatively rigid core composed of the AAA+ domains, while the smaller peripheral regions, namely NTDs and adapter proteins, exhibit pronounced flexibility with respect to this core. In consensus reconstructions, these peripheral regions, often those of particular interest, are averaged out at the expense of the stronger AAA+ density. Thus, the mechanisms of adapter interaction are frequently obscured. To overcome these challenges, many algorithms have been developed to improve visualization of peripheral or flexible regions (90). Most prominent among these are focused classification and refinement, which allow the user to focus on a region of interest by using a mask around the desired density (90). These strategies have been employed successfully in several studies of p97 or its homologs (23, 24, 56).

**Figure 5:**
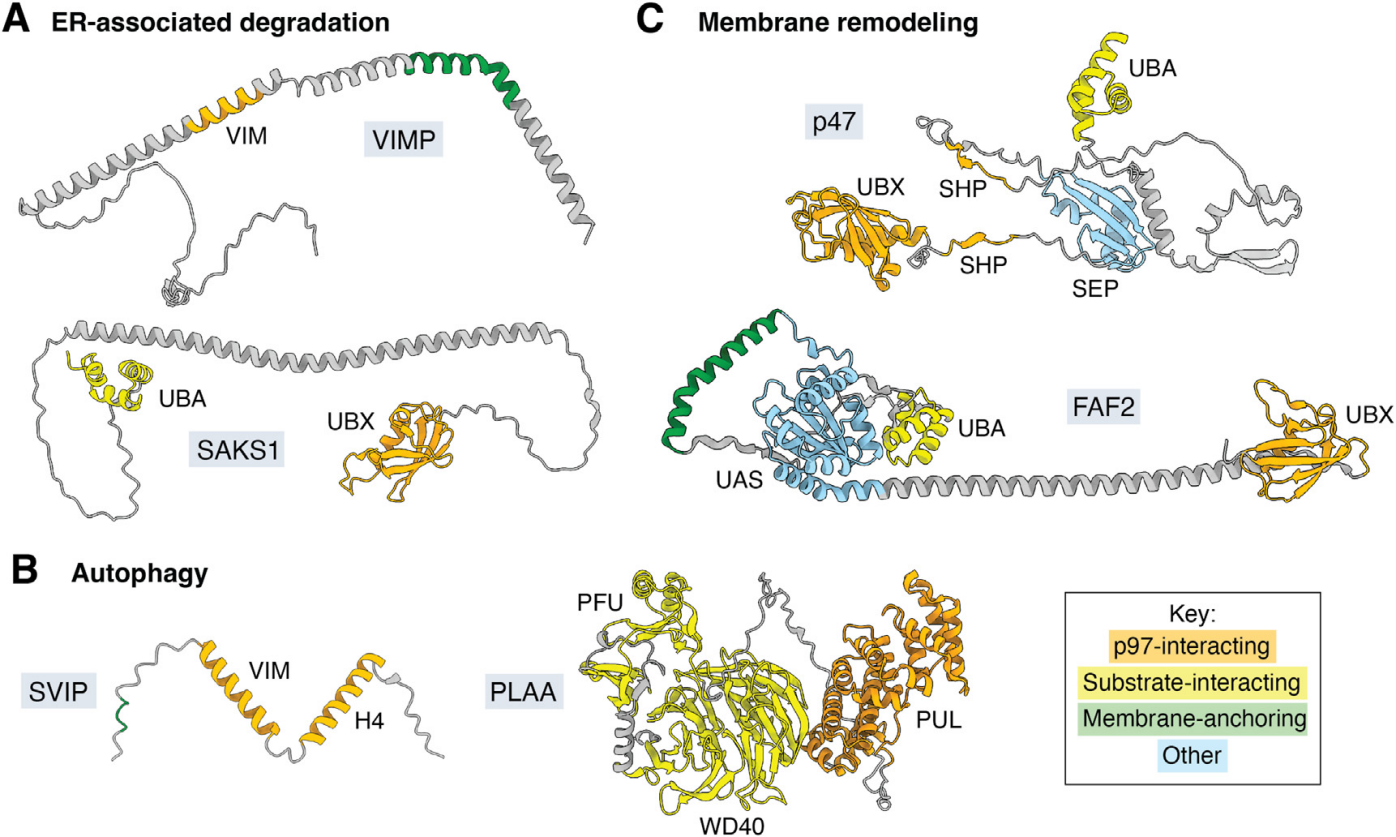
**AlphaFold2 predictions of selected p97 adapters.** AlphaFold2 models of p97 adapters implicated in (*A*) ER-associated degradation, (*B*) membrane remodeling, and (*C*) autophagy. Domain colors are indicated by the key. ER, endoplasmic reticulum.

Accurate, high-throughput protein structure prediction is also beginning to transform the study of adapters. In particular, with the development of AlphaFold2, RoseTTAFold, and other algorithms, it is now possible to predict the structure of nearly any protein in the human proteome, with many being precomputed and publicly available (91, 92, 93, 94). These predictions have revealed a striking structural diversity of adapters in many p97 pathways, raising many questions about their interactions with p97. Indeed, the (predicted) structures of many adapters suggest novel functions (Fig. 5). For example, the ERAD-related adapter VIMP features a VIM embedded in a much longer helix, which appears to force p97 NTDs into the “up” conformation, possibly to facilitate binding of other adapters (83). SAKS1, also involved in ERAD, is predicted to harbor a similarly long helix, here connecting substrate- and p97-binding domains (UBA and UBX, respectively). This arrangement perhaps implies that precise spatial positioning of these domains is important for SAKS1-enabled functions; however, this has yet to be tested experimentally. The shortest known p97 adapter, the autophagy-related SVIP, features a membrane anchoring domain and two p97-interacting domains, and has been reported to inhibit p97 ATPase activity and ERAD (24, 95, 96, 97), though the molecular basis for these activities is unknown. Finally, the predicted structure of the membrane remodeling-related FAF2 features substrate-binding UBA and membrane-anchoring domains clustered on one end of the protein, and a p97-binding UBX domain at the other. Similar to the predicted structure of SAKS1, these sides appear connected by an extremely long alpha helix, raising key questions about the positioning of these domains with respect to membranes and components of protein complexes. Importantly, structure predictions can also facilitate the identification of unknown densities in experimental structures, as in our study of UBXD1 (24) and a recent study of yeast Shp1 (25). This application is especially important given the large unannotated regions present in many adapters. Further advances in the accurate prediction of multimeric complexes will likely continue to generate hypotheses about p97-adapter interactions.

Complementary to the above structural techniques is the purification of intact p97-adapter complexes from cells or tissues for *in vitro* characterization. Given that many p97-adapter complexes appear fragile or dynamic, isolation from native or native-like environments, followed by cryo-EM and/or proteomic analysis, has the potential to reveal new mechanisms of p97 function and associated interaction partners. This workflow is well-illustrated by studies from the Shen group, in which p97 or Cdc48 complexes actively unfolding substrate were isolated from cultured cells by immunoprecipitation of SEP-domain adapters (56, 85). While these studies focused on the mechanism of substrate translocation, we anticipate that this technique could be expanded to characterize p97 in distinct cellular processes.

Perhaps the most exciting technology that has the potential to deepen our understanding of p97 function is cellular cryo-electron tomography (98). In theory, this technology could allow direct observation of p97 complexes in their native environment at high resolution, which might reveal unknown interacting partners and provide spatial context that appears relevant in many p97-dependent processes. This technology has already been applied to the study of Cdc48 in ERAD in *Chlamydomonas reinhardtii* (99), and it is likely that further developments will enable higher-resolution observations in the future.

## Conclusions and perspectives

The understanding of adapter-mediated p97 regulation gained in the past ∼5 years is substantial, and intriguingly analogous to other proteostasis systems such as the Hsp70-Hsp90 molecular chaperones. Common to these systems is the reliance of a central effector on a network of cochaperone regulators that provide specificity during client processing (100). Additionally, in both systems the roles of these regulators, often not predicted previously, have been illuminated by single-particle cryo-EM studies and other technologies.

Given the functional relevance of many unannotated elements of p97 adapters, it is increasingly apparent that adapter studies *in vitro* are most informative when performed with full-length proteins. This is well-illustrated by studies of UFD1/NPL4, ASPL, and UBXD1, all of which feature unanticipated elements that contribute to p97 binding. However, nearly all other adapters have not been structurally or functionally characterized using full-length proteins, leaving much unknown. A related issue is the disconnect between the molecular basis of adapter interactions with p97 and their associated cellular functions. This is especially true for adapters that do not interact with substrate, as their roles cannot be predicted based on domain architecture alone. Given the complexities of p97-adapter interactions in cells, we anticipate that technologies that bridge the gap between biochemistry and cell biology will enable significant advances in our understanding of these processes.

## Conflict of interest

The authors declare that they have no conflicts of interest with the contents of this article.
